# The impact of targeted lifestyle interventions on brain function in young and middle-aged patients with hypertension: A retrospective cohort analysis

**DOI:** 10.12669/pjms.40.11.10304

**Published:** 2024-12

**Authors:** Yangjie Yu, Qiying Chen

**Affiliations:** 1 Yangjie Yu, Department of Cardiology, Huashan Hospital, Fudan University, Shanghai, 200040, P.R. China; 2 Qiying Chen, Department of Cardiology, Huashan Hospital, Fudan University, Shanghai, 200040, P.R. China

**Keywords:** Lifestyle interventions, Brain function, Young and Middle-aged, Hypertension, Nursing care

## Abstract

**Objective:**

To explore the impact of targeted lifestyle interventions on brain function in young and middle-aged patients with hypertension.

**Methods:**

Clinical data of 104 young and middle-aged patients with hypertension, treated in Huashan Hospital, Fudan University from June 2021 to September 2023, were retrospectively analyzed. Forty nine patients received routine nursing care (control group), and 55 patients received targeted lifestyle interventions in addition to routine nursing care (observation group). Blood pressure levels, cognitive function, memory function, executive function, and working memory ability were compared between the two groups before and after the intervention.

**Results:**

After the intervention, blood pressure levels in both groups decreased compared to before the intervention, and were significantly lower in the observation group compared to the control group (*P*<0.05). After the intervention, both groups showed significant improvements in cognitive function, memory function, executive function, and working memory ability (*P*<0.05).

**Conclusions:**

Targeted lifestyle interventions can significantly improve blood pressure control levels and brain function in young and middle-aged patients with hypertension.

## INTRODUCTION

In recent years, with the increasing exposure to chronic work- and lifestyle-related stress, the incidence of hypertension is on the rise, and the affected population tends to be younger.^1^-^3^ Research shows that there are currently 245 million cases of hypertension in China, and approximately 165 million of them (67.34%) are reported in young and middle-aged patients.^4^,^5^

Hypertension can result in varying degrees of damage to organs such as the kidneys, brain, and heart as the condition progresses.^6^-^8^ Hypertension has become a serious public health issue that significantly impacts quality of life and physical and mental health of patients.^4^-^6^ Current studies indicate that elevated blood pressure may cause changes in brain function and structure at the very early stages of hypertensive disorder, leading to cognitive decline, hypertensive encephalopathy, hemorrhagic stroke, ischemic stroke, small vessel disease.^7^,^8^ Therefore, early intervention is particularly crucial in young and middle-aged patients with hypertension.^9^,^10^

Research has shown that lifestyle habits are closely related to the onset and progression of hypertension,^7^-^9^ and that targeted lifestyle interventions can assist patients in developing good behavioral habits and lifestyles, thus reducing negative impacts of hypertension and ensuring the effectiveness of disease intervention.^10^ Most studies have reported the positive effects of exercise intervention and dietary intervention on cognitive function in patients with hypertension.^11^-^13^ In this study, we also added psychological intervention and sleep intervention to give a broader and more comprehensive lifestyle intervention.

## METHODS

Clinical data of 104 young and middle-aged patients with hypertension, treated in Huashan Hospital, Fudan University from June 2021 to September 2023, were retrospectively analyzed. Patients (n=49) who received routine nursing care were set as the control group, and patients (n=55) who received targeted lifestyle interventions combined with routine nursing care were assigned to the observation group. Patients were considered for inclusion if they met the diagnostic criteria for hypertension,^14^ aged 30 to 40 years old, with three non-daily blood pressure measurements of 90mmHg ≤ DBP < 100mmHg, 140mmHg ≤ SBP < 160mmHg, and had complete clinical data. Exclusions included lactating and pregnant women, or with diabetes, chronic kidney disease, chronic liver disease, malignant tumor, ischemic heart disease, cerebrovascular or psychiatric disorders, secondary hypertension, or limb dysfunction.

### Ethical Approval:

All procedures performed in this study were in accordance with the ethical standards of the institutional and/or national research committee(s) and with the Helsinki Declaration. The informed consent was waived by the ethics committee for the observational and retrospective nature. The ethics committee of Huashan Hospital, Fudan University approved this study with the number 2022-080.

### Routine nursing:

Health education was carried out through organizing health knowledge lectures, distributing health knowledge manuals, and included providing basic knowledge of hypertension, commonly used drugs and common adverse reactions, the importance of standardized medication, daily dietary precautions, and the importance of active exercise.

### Lifestyle interventions:

### Exercise intervention:

Patients were provided moderate intensity exercise intervention. Patients were guided to adopt exercise methods such as Tai Chi, brisk walking, swimming, and jogging, with a duration of 30-45 minutes per session, 15-20 minutes of warm-up, and 3-5 times per week. Patients were instructed to ensure exercise yield>500-1000 MET/minutes/week, with 30-45 minutes per session for various types of moderate intensity muscle training.

### Dietary intervention:

Dietary and nutritional trends based on the patient’s daily energy consumption were analyzed. Focus was made on analyzing daily energy intake of staple and complementary foods. Fat, protein, and carbohydrates intake was moderately regulated according to the three major nutrient standards of the Dietary Guidelines. Sodium intake was controlled, and the intake of potassium and calcium was increased. Consumption of spicy, raw, and cold-stimulating foods was restricted. Patients were advised to quit smoking and drinking and ensure balanced nutritional intake, containing 20% to 25% fat, 45% to 60% carbohydrates, and 15% to 20% protein. Protein was mainly selected from high-quality sources such as fish. Complex sugars were suggested instead of monosaccharides such as sucrose and glucose. Overweight and obese patients were instructed to maintain a “negative balance” energy control system to regulate body mass index and improve metabolic function.

### Psychological intervention:

The communication between nurses and patients was enhanced. Efforts were made to alleviate negative emotions of patients by using encouraging language, describing cases of successful treatment and assisting patients in building treatment confidence and correctly understanding their own diseases through the power of role models. Patients who experienced nervous tension and emotional excitement were guided to take deep breaths and other distractions were used to help regulate their emotional state and prevent negative emotions from stimulating blood pressure.

### Sleep intervention:

Personalized intervention was based on the patient’s sleep habits. Patients were informed of the importance of regular and healthy sleep patterns for good disease outcomes. Patients who had long-term irregular sleep patterns and stayed up late, were guided to change their bad sleep habits, develop the habit of going to bed and getting up early. Patients were instructed to not get up immediately after waking up in the morning, and lie flat for a while before getting up.

### Observation indicators:

- Blood pressure was measured using a dynamic blood pressure monitor (manufacturer: Omron)
- Cognitive function was assessed based on the Mini Mental State Examination (MMSE) and the Montreal Cognitive Assessment Scale (MoCA); The average total score was 30 points, and higher score indicated better cognitive function.^15^
- Memory function was assessed based on auditory word learning test (AVLT) and complex figure test (CFT). Briefly, for AVLT, the researchers read 12 words and asked patients to immediately recall and retell the words they read. Then, the researchers read another 12 words and asked patients to wait for five minutes before retelling. One correct answer was one point, for a total of 24 points. Higher score indicated better memory function. During the CFT, patients were guided to copy a picture. The original was then covered up, and ten minutes later patients were asked to recall and draw a picture on white paper. Accuracy of the drawn graphics was assessed based on the score points and position accuracy. Each correct answer received one point, a total of 48 points. Higher score indicated better memory function.^16^
- Executive function was evaluated based on the Connection Test B (TMT-B) and Stroop Color Word Experiment (SCWT). For TMT-B, different numbers were placed in circles of different colors, and the completion time was recorded based on the order of number size and the connection form of different color intervals. SCWT adopts color reading, with each set testing 50 words and four colors. Patients were required to accurately and quickly state the color of the text and record the time used.^17^
- Working memory ability was evaluated based on the Digital Breadth Test (DST) and the Symbol Number Pattern Test (SDMT). DST is mainly used to evaluate the breadth of attention, including two types of forward and backward repetition. The examiner read a set of numbers and asked the examinee to repeat them as required. The test started with two numbers, and continued with an increasing series of numbers at a speed of one number per second. If the patients passed the level, they advanced to the next one. If the patient failed to reproduce the sequence first time, the test was repeated. If the patients failed both times, the test ended. The number of numbers correctly repeated by the patient determined the score for each item. SDMT included nine encoding keys (different abstract symbols), with each symbol corresponding to a number. Participants were required to quickly (within 90 seconds) write the number corresponding to the symbol. Higher number of correct numbers indicated better attention.^18^

### Statistical Analysis:

Data were analyzed using SPSS version 26.0 (IBM Corp, Armonk, NY, USA). Quantitative data in normal distribution were presented as mean ± standard deviation (SD) and t-test was used to compare two independent samples between groups, while quantitative data in non-normal distribution were presented as median and the interquartile range (IQR) and Mann-Whitney U test was used for comparison between groups. For categorical variables, frequency distribution was provided and expressed as a percentage. The Chi-square test was used to compare categorical variables between two groups, such as gender distribution, education level, and marital status. *P*<0.05 was considered to indicate statistically significant differences. PRISM8.0 software (GraphPad, San Diego, USA) was used to draw bar charts of DBP, SBP, MMSE, MoCA, AVLT, CFT, TMT-B, SCWT, DST, and SDMT before and after patient intervention.

## RESULTS

**Fig.1:**
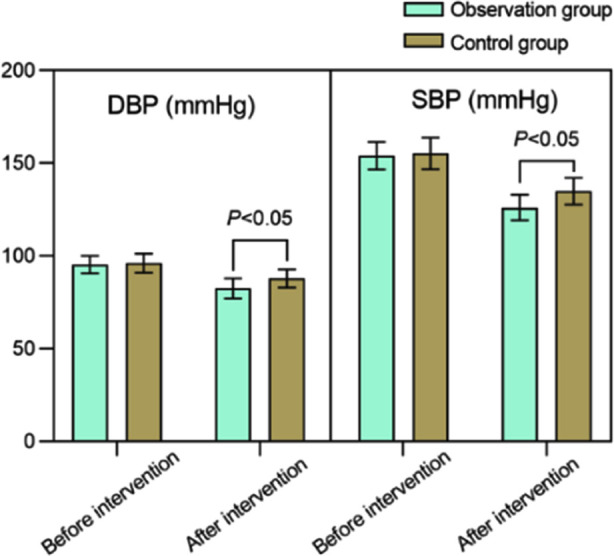
Comparison of blood pressure control levels between two groups of patients; DBP: diastolic blood pressure (DBP); Systolic blood pressure (SBP).

A total of 109 cases met the inclusion criteria for this study, 49 patients in the control group and 55 patients in the observation group. There was no significant difference in baseline characteristics between the two groups (*P*>0.05). Table-I Before the intervention, there was no significant difference (*P*>0.05) between the two groups in the scores of DBP and SBP. After the intervention, DBP and SBP scores in both groups decreased, and were significantly lower in the observation group compared to the control group (*P*<0.05). Fig.1

**Table-I:** Comparison of baseline characteristics between the two groups.

Item	Observation group (n=55)	Control group (n=49)	t/χ^2^	P
***Gender [n (%)]***				
Male	27 (49.09)	28 (57.14)	0.674	0.412
Female	28 (50.91)	21 (42.86)		
Age (year)	44(36,51)	45(36,52)	-0.525	0.599
BMI (kg/m^2^)	25.42±2.70	24.87±2.67	1.043	0.299
***Educational level***				
Below High School	24 (43.64)	25 (51.02)	0.567	0.451
High school and above	31 (56.36)	24 (48.98)		
***Marital status***				
Married	44 (80.00)	42 (85.71)	0.591	0.442
Unmarried/divorced/widowed	11 (20.00)	7 (14.29)		
Disease course (year)	4(3,6)	5(3,6)	-0.713	0.476
LDL-C (mmol/L)	2.50(2.00,3.20)	2.50(1.60,3.20)	-0.581	0.562
HDL-C (mmol/L)	1.20(0.80,1.40)	1.20(0.80,1.40)	-0.468	0.640
TG (mmol/L)	1.90(1.40,2.50)	1.80(1.30,2.56)	-0.643	0.520
TC (mmol/L)	4.10(3.50,5.10)	4.50(3.50,5.50)	-0.424	0.672

***Note:*** body mass index (BMI); Low-density lipoprotein cholesterol (LDL-C); High-Density Lipoprotein Cholesterol (HDL-C); Thyroglobulin (Tg); total cholesterol (TC).

**Fig.2:**
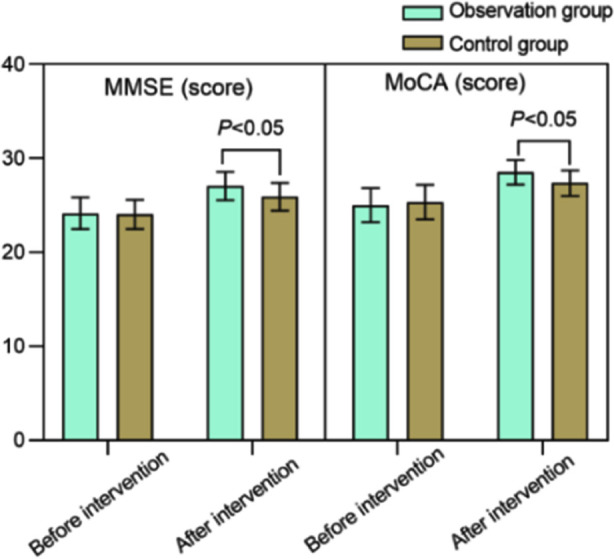
Comparison of MMSE and MoCA scores between two groups; Mini-Mental State Examination (MMSE); Montreal Cognitive Assess.

**Fig.3:**
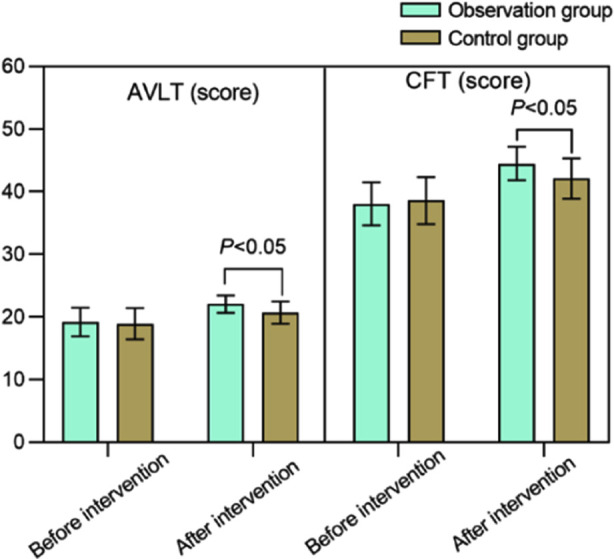
Comparison of AVLT and CFT scores between two groups; Rey Auditory Verbal Learning Test (AVLT); Complex Figure Test (CFT).

Before the intervention, there was no significant difference in MMSE and MoCA scores between the two groups (*P*>0.05). Intervention led to increase in MMSE and MoCA scores in both groups increased, and the post-intervention scores were significantly higher in the observation group compared to the control group (*P*<0.05). Fig.2 Before the intervention, AVLT and CFT scores were comparable in the two groups (*P*>0.05). After the intervention, AVLT and CFT scores of both groups increased, and were significantly higher in the observation group than in the control group (*P*<0.05). Fig.3

**Fig.4:**
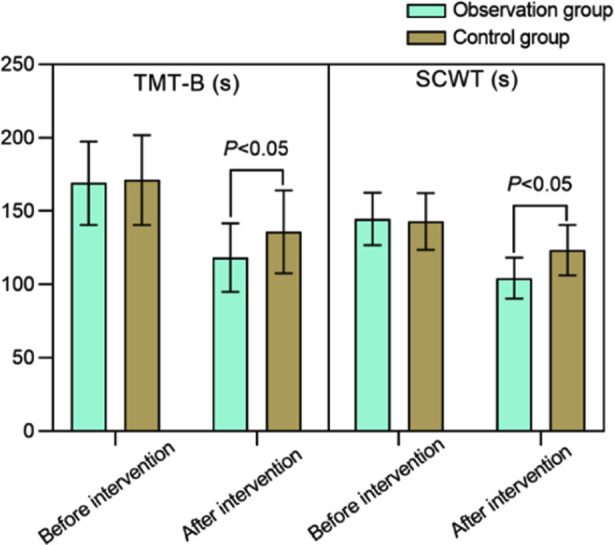
Comparison of TMT-B and SCWT scores between two groups; Trail Making Test Part-B (TMT-B:); Second (S).

**Fig.5:**
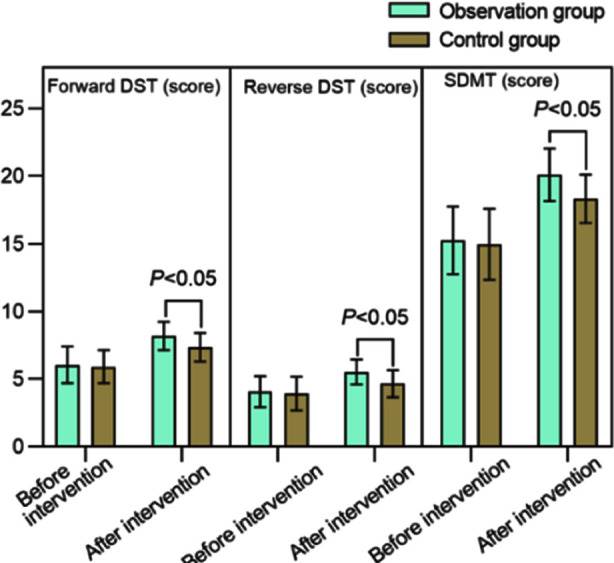
Comparison of DST and SDMT scores between two groups; Digital Span Test (DST); Symbol Digit Modalities test (SDMT).

There was no significant difference in the pre-intervention duration of TMT-B and SCWT in both groups (*P*>0.05). After the intervention, duration of TMT-B and SCWT in both groups decreased compared to before the intervention, and was markedly lower in the observation group (*P*<0.05). Fig.4 Similarly, DST and SDMT scores were similar in both groups before the intervention (*P*>0.05), but increased significantly after the intervention, and patients in the observation group had markedly higher post-intervention DST and SDMT scores compared to patients in the control group (*P*<0.05). Fig.5

## DISCUSSION

This study showed that lifestyle interventions can improve the blood pressure control effect in the populations of patients with hypertension. Our observations confirmed previous reports, which have also been studied from multidomain of lifestyle.^10^,^19^,^20^

Studies have shown that adopting good behavioral habits and healthy lifestyle can relieve overall stress, alleviate depression and anxiety, improve glucose and lipid metabolism, suppresses central nervous system excitability, relieve small artery spasms, and reduce peripheral vascular resistance. All these factors promote a gradual return of blood pressure to normal.^21^-^23^ Alves et al.^24^ found that standardized exercise training leads to a reduction in peripheral vascular resistance, increase in the heart rate reserve, reduction in carotid artery wall thickness, improvement of endothelial cell function, and reduction in the risk of cerebrovascular complications. Furthermore, active exercise can reduce blood viscosity, regulate the sensitivity of the sympathetic nervous system, inhibit norepinephrine production, and promote vasodilation.^22^-^24^

Our study confirms that targeted lifestyle interventions have a marked positive effect on the brain function in young and middle-aged patients with hypertension, and are associated with an improvement in cognitive function, memory, and executive abilities. Animal studies by Lee et al.^25^ showed that aerobic exercises can improve cognitive function in hypertensive mice. Hypertension can damage cerebrovascular structure and cause atherosclerosis^26^ that may impact cerebrovascular function, and cause cognitive impairment.^25^,^26^ A study by Teixeira et al.^27^ demonstrated that lifestyle interventions such as active exercise intervention can help improve blood pressure levels and cognitive function in patients with hypertension, and suggested out that this effect of lifestyle interventions is due to the increased synthesis of nitric oxide in endothelial cells that promote endothelial relaxation, lower blood pressure, and reduce the damage of hypertension to brain tissue and structures. Positive healthy behavior can enhance arterial pressure reflex, activate the aortic arch and carotid sinus reflex center, enhance the excitability of the vagus nerve center, increase vagus nerve efferent activity, and reduce sympathetic nerve efferent activity.^26^-^28^ Standardized exercise training can improve overall metabolism, reduce oxidative stress damage, promote nitric oxide generation by endothelial cells, improve oxygen supply, relax blood vessels, regulate blood pressure, and thus alleviate the damaging impact of hypertension on cognitive function.^24^,and exercise training is recommended by the major professional and scientific societies, including the American College of Sports Medicine (ACSM^25^ John et al.^28^ have confirmed that targeted lifestyle interventions can improve lifestyle and behavioral habits of patients. This type of intervention provides targeted guidance based on patient’s exercise and dietary preferences, and daily habits. Such tailored interventions promote better adherence of patients to the program and improves their willingness to follow medical advice and maintain a healthy lifestyle, thereby improving disease control effectiveness.

Smith et al.^12^ also found that patients with hypertension have a certain degree of cognitive impairment, which is closely related to their lifestyle habits. By implementing comprehensive management of such patients through lifestyle interventions, clinicians may directly address unhealthy habits such as lack of exercise and high salt and sodium diet that exacerbate hypertension and lower the effectiveness of blood pressure control measures. Appropriate exercise, adherence to medical standards for medication, and a healthy diet can improve physical fitness, enhance blood pressure control, and alleviate the damage of hypertension on cerebrovascular structures, thereby achieving the goal of improving cognitive function.^27^,^28^ Our study further confirms that lifestyle intervention can control blood pressure and improve brain function of young and middle-aged patients with hypertension.

### Limitations:

Firstly, this is a single center retrospective study with small sample size and possible selection bias. Secondly, no follow-up analysis was conducted, and a longer follow-up period is needed to verify the results. Thirdly, there are differences in the living conditions and lifestyle of patients, which may have a certain impact on the implementation of intervention measures. Finally, the impact of lifestyle interventions on long-term brain function recovery in patients was not analyzed. Further higher quality studies are needed to verify our conclusions.

## CONCLUSION

Targeted lifestyle interventions can improve blood pressure control and brain function of young and middle-aged patients with hypertension.

### Authors’ contributions:

YY: Conceived and designed the study. Manuscript Writing, Critical Review.

YY and QC: Collected the data, performed the analysis, Critical Review..

All authors have read and approved the final manuscript and are also responsible for the integrity of the study.
